# Decellularization of human stromal refractive lenticules for corneal tissue engineering

**DOI:** 10.1038/srep26339

**Published:** 2016-05-23

**Authors:** Gary Hin-Fai Yam, Nur Zahirah Binte M. Yusoff, Tze-Wei Goh, Melina Setiawan, Xiao-Wen Lee, Yu-Chi Liu, Jodhbir S. Mehta

**Affiliations:** 1 Tissue Engineering and Stem Cell Group, Singapore Eye Research Institute, Singapore; 2 Eye-ACP, Duke-NUS Graduate Medical School, Singapore; 3 Singapore National Eye Center, Singapore; 4 School of Materials Science and Engineering, Nanyang Technological University, Singapore

## Abstract

Small incision lenticule extraction (SMILE) becomes a procedure to correct myopia. The extracted lenticule can be used for other clinical scenarios. To prepare for allogeneic implantation, lenticule decellularization with preserved optical property, stromal architecture and chemistry would be necessary. We evaluated different methods to decellularize thin human corneal stromal lenticules created by femtosecond laser. Treatment with 0.1% sodium dodecylsulfate (SDS) followed by extensive washes was the most efficient protocol to remove cellular and nuclear materials. Empty cell space was found inside the stroma, which displayed aligned collagen fibril architecture similar to native stroma. The SDS-based method was superior to other treatments with hyperosmotic 1.5 M sodium chloride, 0.1% Triton X-100 and nucleases (from 2 to 10 U/ml DNase and RNase) in preserving extracellular matrix content (collagens, glycoproteins and glycosaminoglycans). The stromal transparency and light transmittance was indifferent to untreated lenticules. *In vitro* recellularization showed that the SDS-treated lenticules supported corneal stromal fibroblast growth. *In vivo* re-implantation into a rabbit stromal pocket further revealed the safety and biocompatibility of SDS-decellularized lenticules without short- and long-term rejection risk. Our results concluded that femtosecond laser-derived human stromal lenticules decellularized by 0.1% SDS could generate a transplantable bioscaffold with native-like stromal architecture and chemistry.

A transparent and highly refractive cornea is crucial for vision. Refractive errors are the commonest eye disorders and they can be corrected by glasses, contact lenses or through refractive surgery. Laser-assisted *in situ* keratomileusis (LASIK) has been widely used to treat myopia, hyperopia and astigmatism^1^,^2^. Since 2011, small incision lenticule extraction (SMILE) has become clinically available in Europe and Asia as an alternative to LASIK^3^,^4^. Until now, over 380,000 SMILE procedures have been conducted worldwide (data from Carl Zeiss Meditec). The procedural numbers are still small compared to LASIK, but the trend is uprising. With increasing number of patients undergoing SMILE, the extracted piece of stromal lenticule (refractive lenticule; with an average diameter of 6.5 mm) could be used for other ophthalmic scenarios^5^,^6^. Lenticule re-implantation has been shown to restore the stromal volume and refractive error indices after myopic ReLEx (refractive lenticule extraction) in rabbit and monkey models^7^,^8^. Corrections of hyperopia and keratoconus in patients by lenticule implantation have also been reported^9^,^10^,^11^. Although the predictability is yet to be proven, this procedure seems to be clinically safe and effective.

Lenticule implantation maybe performed in an autologous manner, however, in reality, the majority of cases would be allogeneic especially if they are being used for hyperopic correction or therapeutically in keratoconus. Though the cornea is an immune privileged tissue, stromal allograft rejection can still occur. A recent meta-analysis study showed 3 to 24% graft rejection rate in patients receiving deep anterior lamellar keratoplasty (DALK), depending on the underlying diseases^12^. Topical corticosteroids can be effective to reverse the stromal edema and recover visual functions, however the patients are at risk to have intraocular pressure rise and cataract. To eliminate these potential hazards, the implanted lenticule needs to be non-immunogenic.

Residual cellular materials in allograft can influence host-graft tissue integration, which might lead to graft rejection^13^,^14^. Potential triggering factors are the cell membrane epitopes, allogeneic DNA and a group of damage-associated molecular pattern (DAMP) molecules, which could be removed by proper decellularization^15^,^16^. As most decellularization procedures are shown to have negative effects on extracellular matrix (ECM), this may compromise the biomechanical and optical properties of stromal lenticules and hamper their potential corneal applications.

Different protocols using chemical, enzymatic or physical means to decellularize human and animal corneal tissues have been reported^17^. Acids and alkali can solubilize cytoplasmic and nuclear components but will denature collagen fibrils and proteoglycans, resulting in poor ECM strength. Detergents are effective in dissolving cell membrane and DNA-protein interactions. Triton X-100 (Tx, non-ionic detergent) targets on lipid-lipid and lipid-protein interactions, but is ineffective to remove cellular materials^18^. Sodium dodecylsulfate (SDS, ionic detergent) efficiently solubilizes cellular components, however conflicting data have shown its consequences on ECM disruption^19^,^20^,^21^,^22^. As such, further characterization is necessary to verify its effect on ECM preservation. Enzymes, like trypsin and dispase, can disrupt ECM and remove collagens and glycosaminoglycans (GAGs). Nucleases (DNase and RNase) breakdown nucleic acids but the products are difficult to be cleared from ECM, and may invoke immune response, impeding recellularization and translational usage^17^. Physical methods, like freezing and thawing, pressurization and voltage, have direct impact on ECM structure and are expensive to perform. In view of these variable results, we performed a comprehensive comparison of various protocols and optimized a feasible method to decellularize thin human stromal lenticules. Our work was aimed to generate acellular thin lenticules with preserved transparency and stromal architecture as a bioscaffold suitable for recellularization and stromal implantation.

## Results

### Optical efficiency of treated lenticules

**Figure 1:**
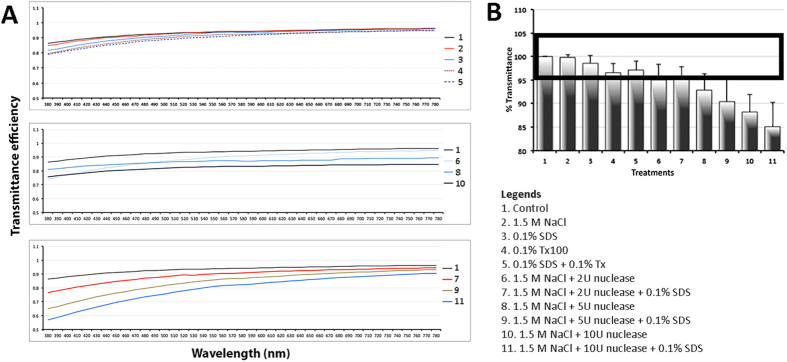
Optical properties of decellularized lenticules. (**A**) Graphs showing mean spectral transmittance at wavelengths ranging from 380 to 780 nm. Y-axis is the normalized transmittance coefficient (C_t_). (**B**) Comparison of mean percentage of Ct along the transmittance spectrum denoted that 6 treatments had similar transmittance as control lenticules. Bold rectangle represents transmittance levels within 5% (95 to 105%) compared to control (insignificant variation).

After treatment using various decellularization protocols and deturgescence with glycerol, the lenticules were first analyzed for the spectral transmittance (380 to 780 nm wavelength). All treatments resulted in reduced transmittance compared to control lenticules (Fig. 1A). In general, the reduction was more pronounced for near-ultraviolet wavelengths, with the exception for the global group of nuclease without SDS treatment. The mean percentage of transmittance coefficient (Ct) of all 41 spectral points (at 10 nm intervals) was calculated for different treatments, each performed in triplicate. Treatments of 1.5 M NaCl, 0.1% SDS, 0.1% Tx, and 2 U/ml nuclease with or without 0.1% SDS showed insignificant transmittance changes (<5%) as compared to control (bold rectangle in Fig. 1B). Among them, the best transmittance close to the control was NaCl, followed by 0.1% SDS treatment group.

### Lenticule thickness

**Figure 2:**
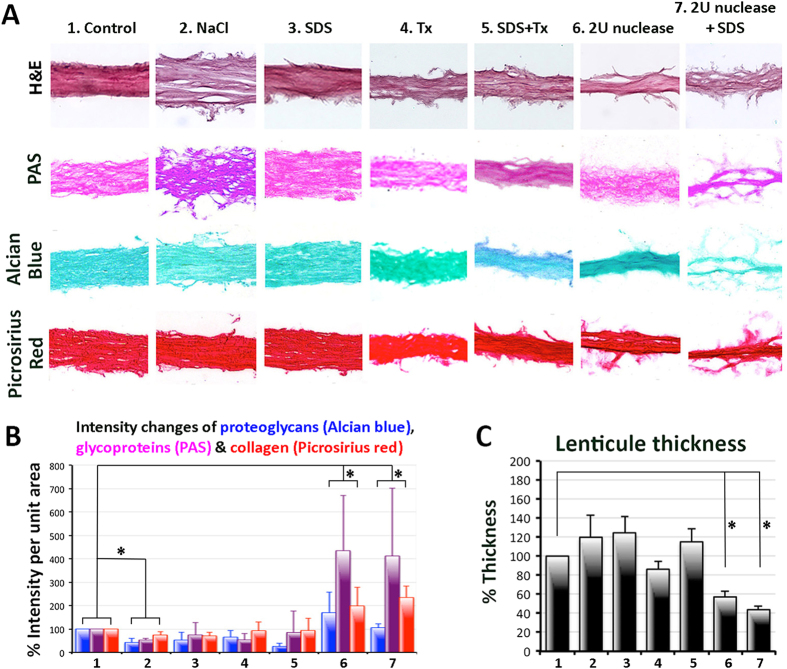
Lenticule morphology and non-fibrillar extracellular matrix. (**A**) Representative histochemistry pictures of lenticules after decellularization by 6 selected methods from transmittance assay. The lenticules were stained by hematoxylin-eosin (H&E), periodic acid-Schiff (PAS), alcian blue and picrosirius red. (**B**) Histogram of the mean staining intensity and SD. *P < 0.05 for multiple comparison of all 3 non-fibrillar components between control and treated lenticules. (**C**) Histogram showing lenticule thicknesses. Data are presented as mean and SD and compared to untreated control (100%). Nuclease treatment resulted in significantly reduced thickness (*P < 0.05, Mann-Whitney U test).

On hematoxylin-eosin (H&E) stained sections, the lenticule thickness was measured at 5 standard positions and the mean percentages of thickness of treated lenticules were calculated with reference to control. Treatments with 1.5 M NaCl, 0.1% SDS and 0.1% Tx showed mild thickness changes (Fig. 2C). Nuclease treatments appeared to result in lenticules of significantly reduced thickness (Fig. 2C and Supplementary Fig. S1). This could be related to the extensive loosening of fibrils from the lenticule (H&E image in Fig. 2A), resulting in rough surface and inconsistent stromal composition.

### Quantitative evaluation of extracellular matrix components

The stromal content was evaluated by component-based histochemistry. Changes of global collagen were assessed by the staining of Picrosirius red, glycoproteins by PAS and glycosaminoglycans by Alcian blue methods (Fig. 2A). Quantification analysis of staining intensity showed that NaCl, SDS and Tx treatments gave a more homogeneous collagen and proteoglycan content and the values were similar to untreated control (100%) (Fig. 2B). The collagen signal was 74 ± 13% for NaCl (*P* = 0.18, compared to control; Mann-Whitney U test), 71 ± 15% for SDS (*P* = 0.056) and 93 ± 36% for Tx (*P* = 0.076), respectively (Table 1). Similarly, the GAG signal after SDS, Tx and NaCl treatments were 53 ± 33% (*P* = 0.095), 65 ± 30% (*P* = 0.16) and 42 ± 19% (*P* = 0.53), respectively. For glycoproteins, SDS-treated lenticules showed similar staining intensity (75 ± 52%) as control. NaCl and Tx treatments gave slightly reduced glycoprotein content (53 ± 19% for NaCl and 53 ± 28% for Tx).

Nuclease treatments generally resulted in uneven staining pattern and had significantly elevated staining intensities of collagen (Fig. 2A). These were 198 ± 81% for 2 U/ml nuclease (*P* = 0.076, compared to control; Mann-Whitney U test) and 234 ± 49% for 2 U/ml nuclease + SDS (*P* = 0.025) (Fig. 2B; Table 1). The same nuclease treatments also significantly changed glycoprotein intensity. It was 435 ± 66% for 2 U/ml nuclease (*P* = 0.027) and 412 ± 89% for 2 U/ml nuclease + SDS treatment (*P* = 0.011). Similar results were observed for GAGs, which had denser and inconsistent staining pattern (170 ± 88% for 2 U/ml nuclease and 106 ± 15% for 2 U/ml nuclease + SDS treatment). Other treatments with 5 or 10 U/ml nuclease with or without 0.1% SDS also gave inconsistent histology results and significantly altered ECM component contents as compared to control (Supplementary Figs S2 and S3; Table 1).

Multiple comparisons were performed to analyze all three components (collagens, glycoproteins and GAGs) between control and treatment groups using Kruskal Wallis test with post-hoc Dunn-Bonferroni correction. Results showed that treatments with 0.1% SDS, 0.1% Tx and combined SDS/Tx had no statistical difference compared to untreated control (Table 1). In contrast, lenticules treated with hypertonic 1.5 M NaCl and/or nucleases (2 to 10 U/ml) had significantly varied global ECM content (Supplementary Fig. S3).

### Decellularization and denucleation efficiency

**Figure 3:**
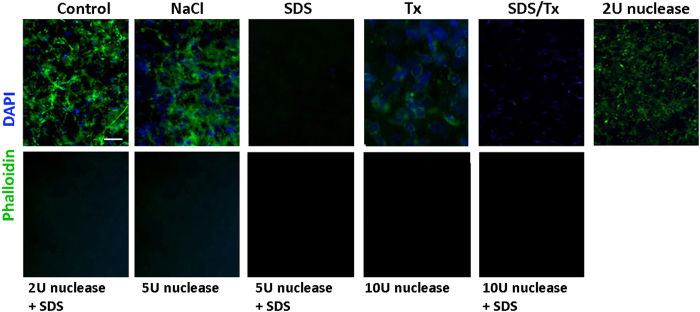
Residual cellular and nuclear materials in lenticules. Representative immunofluorescence pictures showing phalloidin (for cellular actin) and DAPI (for nuclear materials) in control and decellularized lenticules. Scale bar: 50 μm.

The efficiency of eliminating both cellular and nuclear materials was assessed by staining with phalloidin (for intracellular F-actin) and DAPI (for nucleic acid). When compared to control lenticules, all treatments had reduced phalloidin signals (Fig. 3). The treatment with SDS resulted in drastic depletion of cellular materials. This was corroborated by the results after SDS/Tx treatment when compared to Tx only, or after the combined nuclease and SDS treatment compared to nuclease only. In contrast, Tx did not clearly remove cellular and nuclear residues. Treatment of 1.5 M NaCl with or without 2U nuclease also showed the presence of cellular residues. DAPI signals were detected in NaCl- and Tx-treated samples, but not after SDS and nuclease treatments. DNA extraction from SDS-decellularized lenticules revealed significantly reduced residual DNA (20.71 ± 4.3 ng DNA per mg dry weight), compared to control lenticules (298.6 ± 75.01) (*P* = 0.042; paired Student’s t-test). Agarose gel electrophoresis further revealed that the extracted DNA fragments from SDS-treated lenticules were predominantly less that 200 bp in size (Supplementary Fig. S4). NaCl-treated lenticules not only showed DNA fragments with size around 200 bp, but also supercoiled DNA, corroborating with our DAPI staining result.

As a whole, 0.1% SDS treatment was superior among various methods in this study with complete elimination of cellular and nuclear debris, and it was sufficient to maintain stromal collagen, glycoprotein and GAG contents.

### Fibrillar ultrastructure

**Figure 4:**
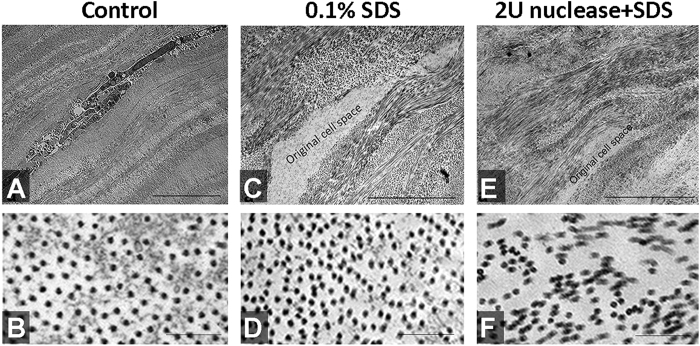
Fibrillar structure of lenticules. (**A**,**B**) Control lenticule, (**C**,**D**) 0.1% SDS-treated lenticule and (**E**,**F**) 2 U/ml nuclease and 0.1% SDS-treated (2N + SDS) lenticule at low and high magnifications. (**A**,**C**,**E**) Transmission electron micrographs at lower magnification showing the global collagen fibril pattern and empty cell space after lenticule treatments by SDS and 2N + SDS, when compared to control. More regular fibril organization was observed in control and SDS-treated lenticules, whereas uneven compaction and irregularities were seen in 2N + SDS lenticule. Scale bars: 5 μm. (**B**,**D**,**F**) Images at high magnification showing the cross-sectional view of collagen fibrils. Scale bars: 200 nm.

In this experiment, lenticules collected at anterior one-third of the corneal stroma (at depth from 180 to 280 μm from the anterior surface) were used for decellularization treatments and control. The lenticules were processed for TEM morphology and morphometric analyses. Intact stromal keratocytes were present within the collagen fibrils in control lenticules (Fig. 4A). In contrast, SDS-treated lenticules contained empty space devoid of cells or cellular debris (Fig. 4C). The ECM organization was similar between SDS-treated and control lenticules. The collagen fibrils were evenly arranged without any distortion (Fig. 4B,D). Measurements of 10 randomly selected regions (fixed window size of 0.5 μm × 0.5 μm) in 3 images showed the mean fibril density of SDS-treated lenticules (729 ± 66/μm^2^) was similar to control lenticules (676 ± 66/μm^2^) (*P* = 0.29, Mann-Whitney U test). On the contrary, treatment with 2 U/ml nuclease + SDS created collagen fibril alterations (Fig. 4E). Higher magnification showed the fibril pattern was greatly distorted (Fig. 4F).

### *In vitro* recellularization and cytotoxicity

**Figure 5:**
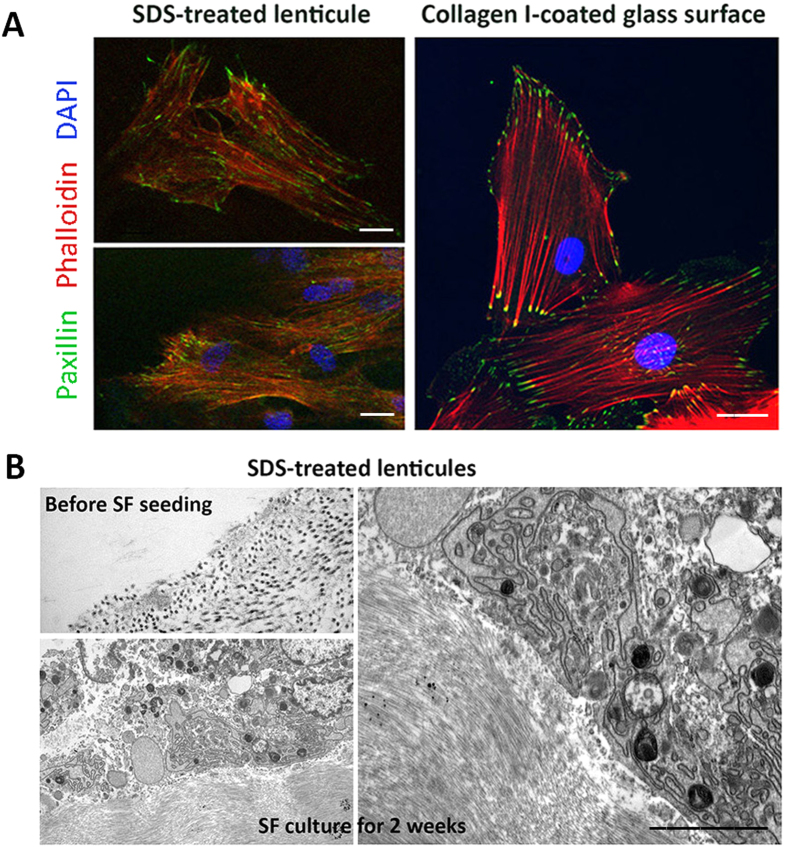
Recellularization on SDS-decellularized lenticules. (**A**) Confocal immunofluorescence showed similar expression of paxillin (focal adhesion complex protein) and phalloidin (F-actin fibers) in human stromal fibroblasts cultured on SDS-treated lenticules and collagen I-coated glass surface for 72 hours. Scale bars: 10 μm. (**B)** TEM micrographs showing SDS-treated lenticules before cell seeding and after culture with stromal fibroblasts for 2 weeks. Cells were closely adhered on the lenticule surface and did not show any signs of cellular toxicity, including dilated endoplasmic reticulum (ER) and induction of lysosomes and intracellular vesicles. Scale bar: 0.5 μm.

Human corneal stromal fibroblasts were cultured on SDS-treated lenticule surface for 72 hours. Live/dead viability assay showed the viable cell count was 91 ± 4.5%. This was comparable to cells on collagen I-coated glass surface (97 ± 2%). Immunofluorescence of paxillin and phalloidin revealed similar focal adhesion patterns (Fig. 5A). Paxillin was located along cell periphery and interacted with F-actin at plasma membrane. TEM images for cells after 2-week culture illustrated a close adherence of cells on SDS-treated lenticule. There was no sign of toxicity as demonstrated by the absence of dilated endoplasmic reticulum (ER) and no clear induction of lysosomes and intracellular vesicles (Fig. 5B).

### *In vivo* assessment of SDS-decellularized lenticules

**Figure 6:**
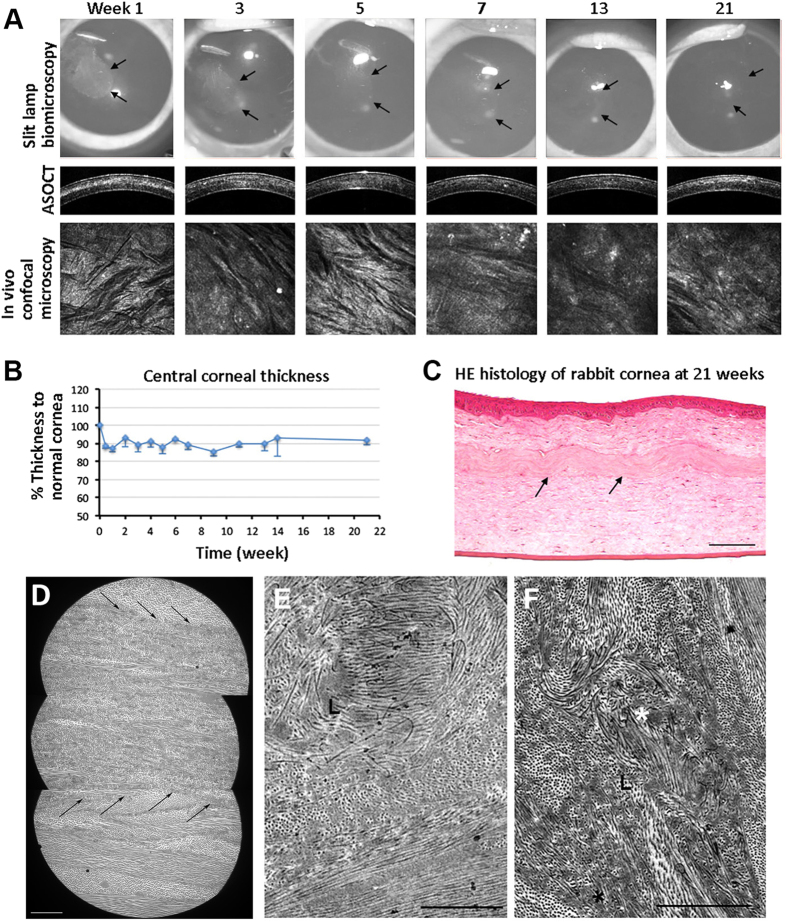
Re-implantation of SDS-decellularized human lenticules to rabbit corneal stromal pockets. (**A**) Ophthalmic examination of corneas with implanted lenticules at different weeks. Gray-scale slit lamp images showed a slight opacity of the implanted lenticule at week 1 and the transparency was improved afterwards. ASOCT displayed a demarcation line with increased reflectivity at the lenticule plane at week 1 and it became less distinct afterwards. *In vivo* confocal microscopy showed a lack of highly reflective cells at the lenticule anterior plane at early weeks and some reflective spots at week 21. (**B**) Percentage changes of central corneal thickness of lenticule-implanted corneas compared to normal corneas. (**C**) H&E histology of cornea with lenticule implantation for 21 weeks. Lenticule was integrated with host stromal tissue. Cell-like structures were seen at the lenticule edge (arrows). No inflammatory cells were observed. Scale bar: 100 μm. (**D**) TEM images showing compact fibrillar structure of implanted lenticule and it was integrated with host stroma (arrows). Scale bar: 10 μm. (**E**) Collagen fibrils inside lenticule displayed irregular orientation and less distinct lattice arrangement. (**F**) Radial arrangement (*) of collagen fibrils inside lenticule. Scale bars: 2 μm.

We assessed the short- and long-term corneal response after implantation of SDS-decellularized human lenticule into corneal stromal pockets of 6 rabbits. There was minor haze at the site of lenticule implant at 1 week post-transplantation but the clarity improved after 2 weeks and the corneas remained clear thereafter (Fig. 6A). No rabbit showed complications, e.g. necrosis, neovascularization, inflammation and extrusion. Two rabbits died at 4^th^ and 7^th^ week, due to anesthetic and sedative-related reasons. Evaluation using ASOCT revealed distinct demarcation of the implanted lenticule at 1 week post-transplantation, which became indiscernible at later time points (Fig. 6A). The central corneal thickness was maintained consistent at about 90% of the contralateral normal corneas (Fig. 6B). This is explained by performing −6.00 diopter SMILE on the rabbit cornea (about 320 μm thick) and this accounted for an extraction of 90 μm lenticule. Subsequent implantation of human acellular lenticule (70 μm thick) would result in a slightly thinner cornea, which was about 90% as normal cornea. *In vivo* confocal microscopy showed the rabbit cornea and lenticule remained quiescent (Fig. 6A). There was a lack of hyper-reflective keratocytes in the implanted lenticule plane at early time points. At 21^st^ week, some hyper-reflective signals were seen. Histology showed hematoxylin-stained nuclei present at the interface between host stroma and implanted lenticule (arrows in Fig. 6C). The corneal layers were intact with a close alignment of implanted lenticule within the host stroma. Under TEM, the implanted human acellular lenticule appeared with more compact fibrillar structure and well integrated with the host stroma without any space in between the host and implanted tissues. In some regions, this demarcation was not discernable (arrows in Fig. 6D). Cell structures were rarely found within the lenticule. At higher magnification, the collagen fibril profile of implanted lenticule was different from that of the host stroma with irregular, fusiform orientation, and less distinct lattice arrangement (Fig. 6E). Empty cell spaces were indistinct and might be re-occupied by collagen fibrils (indicated by white * in Fig. 6F).

## Discussion

In this study, we demonstrated that thin human stromal lenticules were optimally decellularized by 0.1% SDS followed by extensive buffer washes. The resultant lenticules were transparent and the stromal matrix, both fibrillar structure and non-fibrillar components, was similar to native lenticules. The corneal stromal fibroblasts grown on SDS-decellularized lenticules were viable and had negligible cytotoxicity. Implantation of decellularized human lenticule into a rabbit corneal stromal pocket did not elicit any short- or long-term immune response even though corticosteroid administration was stopped after the first 3 days post-surgery. The implanted lenticules integrated well with the host tissue and remained clear in rabbit eyes for more than 5 months. Taken together, the decellularization of thin human stromal lenticules using 0.1% SDS is a plausible approach in generating a suitable bioscaffold for stromal tissue engineering.

In this optimization study, instead of using clinical SMILE samples of variable thickness, we customized stromal lenticules with constant thickness (70 μm) to reveal the treatment effects on stromal structure and transmittance. Among various methods, 0.1% SDS treatment for 24 hours under agitation followed by extensive washes decellularized the lenticules with minimal disturbance to the stromal ECM structure and protein content. There were no gross changes of lenticule shape and thickness after SDS decellularizaiton, unlike nuclease treatment. More extensive investigations using rheometry would be required to study lenticule biomechanics^23^. The efficiency of decellularization was evidenced by the negligible phalloidin signal together with emtpy cell space under TEM. The residual DNA was effectively diminished to ~6.7% of untreated stroma or ~20 ng DNA per mg stromal dry weight, which fulfilled the standard requirement of less than 50 ng DNA per mg dry weight^17^. The fragmented DNA were shorter than 200 bp in length, which would be unlikely to elicit adverse tissue remodeling response^24^. Growth of corneal stromal fibroblasts on SDS-decellularized lenticule further demonstrated that the protocol had negligible cytotoxicity. The cell-matrix adhesion, as shown by paxillin expression, appeared similar to cells grown on regular culture surface. We recellularized the acellular lenticules with corneal stromal fibroblasts, which were the proliferating stromal keratocytes under serum culture, to elucidate if the decellularization would affect the lenticule structure and chemistry for the new keratocytes to reside once it was implanted *in vivo*. The collagen fibril alignment assessed by fibril density was similar to the native stroma. However, the lenticules treated with combined nuclease (2 U/ml DNase and RNase) and 0.1% SDS displayed disorganized fibrillar pattern (Supplementary Fig. S5). In addition, nuclease treatment (from 2 to 10 U/ml) gave an altered histological profile with stromal fibrils loosened from the matrix core, resulting in rough structure and inconsistent stromal architecture and ECM composition. In general, the thin lenticules became thinner and more compact after nuclease treatments, giving a higher intensity range of various ECM components (collagens, glycoproteins and GAGs). Shafiq *et al*. described the nuclease treatment (5 U/ml DNase and RNase) to decellularize full thickness human corneas, which subsequently supported the growth of corneal epithelial cells^21^. Decellularization of whole cornea with basement membrane on both sides may restrict fibril distortion. Yoeruek *et al*. reported a complex protocol of hypotonic buffer with EDTA, aprotinin, SDS and bovine nucleases for the decellularization of whole bovine corneas^25^. In our experiments, we employed a simpler protocol with 0.1% SDS to decellularize thin human stromal lenticules. Similar SDS effect had been reported to decellularize whole porcine corneas^26^. In contrast to the latter results where more than 20% residual cells were present after 0.1% SDS treatment for 24 hours, we were able to achieve better decellularization efficiency. This was probably due to the thin lenticules without Bowman’s and Descemet’s membranes in restricting the reagent permeation and cell removal. Recently, Wilson *et al*. showed that 0.5% SDS efficiently decellularized human whole corneas after long-term storage, however it greatly compromised the stromal matrix structure and transparency (measured at 480 nm wavelength under hydrated state)^22^. Our work also detected a similar reduction of transmittance at wavelength near UV range but no difference towards the visible range when compared to control lenticules after dehydration by glycerol. In addition, their immunostaining results showed a greater disruption of non-fibrillar keratan sulfate-GAG inside the treated stroma. This could be due to the SDS denaturation effect on target epitopes resulting in poor detection. In order to avoid this possible artefact, we employed histochemical staining on global GAGs, collagens and glycoproteins, followed by densitometry analysis.

The partially reduced ECM content could be recovered after the repopulation of host stromal cells. In our rabbit implantation experiment, the recipient corneas remained stable and quiet for over 5 months, without any signs of inflammation and rejection. This was similar to a previous report that xenogenic grafting of human stromal sheet to rabbit cornea did not elicit any rejection response^27^. However, the spatial order of collagen fibrils within the lenticule displayed more fusiform-shaped bundles and radial arrangements, suggesting events recapitulating stromal remodeling. This was not a consequence of SDS treatment as we did not detect any collagen fibril derangement in our TEM study of acellular lenticule alone. It could be the host stromal response that the repairing host keratocytes produce enzymes, such as matrix metalloproteinases and collagenases, to reorganize collagen fibrils, however, it requires further investigation. The remodeling process is able to revert to that of the native state but may take months to years^28^.

As the decellularized lenticules is primarily used for stromal reconstruction, it is important to evaluate the optical properties (transparency) and guarantee that the resulting scaffolds have adequate light transmittance capacity. Our study showed that the treatments of 0.1% SDS and 1.5 M NaCl achieved similar transmittance levels as the native stroma. However, NaCl did not effectively remove cells and nuclear materials. The result was different to reports by Oh *et al*. and Gonzalez-Andrades *et al*. describing NaCl decellularization in porcine corneas^20^,^29^. Species difference could be a reason for the discrepancy of treatment outcome. NaCl acts to disrupt DNA-protein interactions and the osmotic shock can lyze cells but is unable to completely remove cellular materials^17^, which was revealed by positive phalloidin and DAPI staining in our study. Also, supercoiled DNA appeared in NaCl-treated lenticules. On the contrary, 0.1% SDS treatment effectively removed cellular and nuclear remnants and had minimal effect on ECM architecture and chemical composition. This contributes to minor optical scattering as compared to native stroma. The mild variation could be due to the partial ECM changes and loss of cells within the stroma in which the cell space is now replaced by a fluid, leading to a minor change of refractive index.

In conclusion, we identified that 0.1% SDS efficiently decellularized thin human stromal lenticules and reproducibly generated acellular lenticules with preserved optical properties, ECM architecture and chemistry, and with recellularization potential. The acellular lenticules had negligible immunogenicity and integrated into the host stroma. This will increase the potential utilization of allogenic lenticules as a bioscaffold for stromal tissue engineering. At present, there is no standard processing, storage and banking protocols for donor stromal lenticules. We anticipate that this decellularized protocol will be beneficial to generate transplantable lenticules for the therapy of corneal stromal defects and could eventually used for different kinds of corneal diseases. Further work should confirm the safety, applicability and treatment outcomes using decellularized lenticules in humans.

## Methods

### Human corneas

Human cadaveric corneoscleral tissues unsuitable for transplantation were obtained from Lions Eye Institute, Tampa, US and transported in Optisol-GS (Bausch & Lomb) at 4 °C. The donor age was ranged from 9 to 71 years old, the death-to-tissue harvest time was less than 24 hours and the time from death to femtosecond laser cut was between 3 to 16 days (Supplementary Table 1).

### Femtosecond laser cutting of lenticules

Human cornea was mounted onto an artificial anterior chamber (AAC) with the epithelial side facing up. The laser application head fitted with a ring adapter was gently placed onto the AAC column. The sample was raised until it was in contact with the Intershield of the laser application head. A lamellar cut with programmed thickness of 70 μm and a 360° side cut were done by Femto LDV Z6 model (Ziemer Ophthalmic), performed by a single surgeon (JSM)^30^. After the laser procedure, the stromal lamella was dissected and the side cut was opened using a Chansue Femto lamellar dissector (Biocon). The lenticule was transferred to a storage medium (Dulbecco’s modified Eagle medium DMEM / F12, 1:1 vol/vol, with 5% fetal bovine serum (FBS), 100 U/ml penicillin G, 100 μg/ml streptomycin sulfate and 1 μg/ml Amphoptericin B; Invitrogen). The laser dissecting procedure was repeated to obtain a series of lenticules with identical thickness (70 μm). Up to 7 pieces of lenticules were collected from one cornea. As there exists a gradation of fibril compactness along the stromal depth (from high density at the anterior stroma to low density at the posterior stroma), the lenticules were labeled according to their vertical positions from the anterior surface. Anterior-most lenticule (with residual epithelium and Bowman’s layer) and posterior-most lenticule (with endothelium and Descemet’s membrane) were discarded from this study. The remaining were stored at −80 °C until experiment.

### Decellularization treatments

After rinses with PBS (0.01 M, First Base), lenticules labeled at similar vertical stromal position were treated with 10 methods (Table 2) or remained as untreated control. The treatments included: (1) hyperosmotic (1.5 M sodium chloride, NaCl, Sigma-Aldrich); (2) detergent (sodium dodecylsulfate, SDS, and Triton X-100, Tx, Sigma-Aldrich) and (3) enzymatic (nucleases with DNase and RNase, Roche). All treatments were done for 24 hours followed by PBS washes for 3 times, each again for 24 hours under agitation (300 r.p.m.). The lenticules were then processed and analyzed as described in the following sections and experiments were done in triplicate. In order to compare the treatment outcome, results were represented as the percentage to the untreated control, unless stated otherwise.

### Optical property

The lenticules were placed in glycerol (Invitrogen) for 2 hours to reduce non-specific tissue swelling prior to optical evaluations^21^. The spectral transmittance of lenticules was determined using INFINITE 200 UV-Vis spectrophotometer (Tecan) with light wavelength ranged from 380 to 780 nm. Lenticules were placed on polystyrene coverslips (Thermanox, Nunc, 13 mm in diameter, 0.2 mm thick) and inserted into the spectrophotometer chamber for transmittance measurement. Data were collected at 10 nm wavelength increments. The transmittance coefficient (Ct) was calculated as C_t_ = I_t_/I_0_, where (I_t_ was the transmitted light intensity and I_0_ was the incident light intensity)^31^. Experiments were triplicated and C_t_ of samples was normalized with the corresponding C_t_ of polystyrene coverslip and the mean percentage of transmittance to the control lenticule group was calculated.

### Immunostaining

Lenticules were fixed with neutral buffered 4% paraformaldehyde (Sigma) and processed for immunostaining^32^. The antibodies were AlexaFluor 488 or 543-conjugated phalloidin (Invitrogen), anti-human paxillin (Millipore) and secondary AlexaFluor 488-conjugated IgG (Jackson ImmunoRes Lab.). Signals were viewed under fluorescence microscopy (AxioImager Z1, Carl Zeiss).

### DNA quantification

Lenticules were dried at 60 °C for 2 hours for dry weight measurement, prior to proteinase K (50 μg/ml, Promega) treatment at 55 °C overnight. The supernatant was mixed vigorously with chloroform in the presence of 1.5 M potassium acetate. After spinning, DNA in aqueous phase was precipitated, dissolved in nuclease-free water and quantified by NanoDrop spectrometry (ND-1000, Thermo Scientific). The DNA fragment size was assessed by 2% agarose gel electrophoresis.

### Histochemistry and quantification analysis

Lenticules were formalin-fixed and processed for paraffin embedding. Sections (4 μm thick) were stained with H&E and photographed using 10x objective (AxioImager Z1). Thickness at 5 standard positions (^1^/_10_, ^3^/_10_, ^5^/_10_, ^7^/_10_ and ^9^/_10_) along the lenticule length was measured using Image J software (NIH) and the mean thickness was calculated. Sections were stained with Picrosirius red (Direct Red 80 kit, Sigma) for global collagens, Periodic acid-Schiff kit (Merck) for global glycoproteins and Alcian blue (Sigma) for global glycosaminoglycans. Under light microscopy, images were taken at 5 standard locations (same as above) under same exposure time, incident light intensity, gamma level and white balance. Staining intensity was quantified using Image J, adjusted with background threshold and calculated as intensity per unit area of lenticule. Results were normalized with the signal intensity of untreated lenticules and expressed as the percentage changes. Of note, lenticules at similar stromal depth were examined in the same round of H&E and three histochemical stainings.

### Transmission electron microscopy (TEM) and morphometry

Lenticules were fixed sequentially with 3% glutaldehyde (EM Sciences), 1% tannic acid (Sigma) and 1% aqueous solution of osmium tetroxide (EM Sciences), and processed for Epon-Aradite embedding^33^. After staining with 3% uranyl acetate (EM Sciences) and lead citrate, ultrathin sections were examined by TEM (JEOL 2100). Cross-sectional view of collagen fibrils was imaged at magnification of 40,000X in 15 random fields from 3 different lenticules. To estimate the fibril density, a window of fixed size (0.5 μm × 0.5 μm) was randomly localized and the number of fibrils was quantified and the fibril density was expressed as the number of fibrils/μm^2^.

### Recellularization

Primary human stromal fibroblasts propagated in DMEM/F12 with 10% FBS and antibiotics were seeded to lenticule surface at a density of 200 cells/mm^2^. Cells were also seeded on collagen I-coated surface as control. They were cultured for 72 hours (for cell viability) and 2 weeks (for TEM). Media changes were performed every 3 days.

### Live Dead assay

Live/Dead^®^ Viability/Cytotoxicity assay (Invitrogen) was used according to manufacturer’s protocol. Calcein AM-stained viable cells and ethidium homodimer-1 stained non-viable cells were quantified under 10x objective, and live/dead cell ratio was calculated.

### Lenticule implantation to rabbits and examination

The protocol (2015/SHS/1034) was approved by the Institutional Animal Care and Use Committee of SingHealth, Singapore. All animals were treated according to guidelines of the Association for Research in Vision and Ophthalmology Statement for the Use of Animals in Ophthalmic and Vision Research. Six New Zealand albino rabbits (8–12 weeks old; 2–2.5 kg body weight) were obtained from Invivos Pte Ltd (Singapore). They were anesthetized with intramuscular injection of ketamine hydrochloride (50 mg/kg, Parnell Lab.) and xylazine hydrochloride (5 mg/kg, Troy Lab.) and the right eyes underwent −6.00 diopter SMILE surgery performed by a corneal surgeon (JSM) using a VisuMax femtosecond laser system (Cal Zeiss Meditec)^30^. After extraction of rabbit lenticule (~90 μm thickness) and irrigation of the stromal pocket with balanced salt solution, the SDS-decellularized 70 μm lenticule was inserted into the pocket and the opening was closed by one 10–0 nylon suture. Rabbit eyes were instilled with 1% prednisolone acetate eyedrops (3 times daily for 3 days), 0.5% proparacaine eyedrops (4 times daily for 2 days) and 0.3% tobramycin eyedrops (4 times daily for 7 days). The suture was removed after one week. Ophthalmic examinations, including slit-lamp biomicroscopy, *in vivo* confocal microscopy using an infrared slit-lamp scanning microscope (Heidelberg Engineering) and anterior segment optical coherence tomography (ASOCT) using RTVue (Optovue Inc.), were performed prior to the procedure, and post-operation every 2 weeks. After 5 months, rabbits were sacrificed by a lethal dose of pentobarbitone injected intravenously, and corneas were harvested for histochemistry and TEM.

### Statistical analysis

All data were expressed as mean and standard deviation (SD). Mann-Whitney U test was used to compare the ranked raw intensity data of each histochemical staining between treatment and control, collagen interfibrillar distance and transmittance. Multiple comparisons of global histochemical staining intensities among treatments were performed using Kruskal–Wallis test with post-hoc Dunn-Bonferroni correction. Statistical analyses were performed using SPSS version 20. *P* value less than 0.05 is considered to be statistically significant.

## Additional Information

**How to cite this article**: Yam, G. H.-F. *et al*. Decellularization of human stromal refractive lenticules for corneal tissue engineering. *Sci. Rep.* **6**, 26339; doi: 10.1038/srep26339 (2016).

## Supplementary Material

Supplementary Information

## Figures and Tables

**Table 1:** Quantitative analysis of non-fibrillar ECM components in decellularized and control lenticules.

	**Treatments**	**Collagens**		**Glycoproteins**		**Proteoglycans**		**Multiple comparison to control**
		**Mean & SD**	***P*** *	**Mean & SD**	***P*** *	**Mean & SD**	***P*** *	P**
1	Control	100%	–	100%	–	100%	–	–
2	1.5 M NaCl	74 ± 23%	0.180	53 ± 19%	0.18	42 ± 19%	0.53	**0.012**^**a**^
3	0.1% SDS	71 ± 25%	**0.056**^**b**^	75 ± 52%	0.151	53 ± 33%	0.095	0.25
4	0.1% Tx	93 ± 36%	**0.076**^**b**^	53 ± 28%	0.18	65 ± 30%	0.16	0.31
5	SDS + Tx	94 ± 53%	0.327	85 ± 43%	0.327	56 ± 33%	0.14	0.18
6	2Nu	198 ± 81%	**0.076**^**b**^	435 ± 66%	**0.027**^**a**^	170 ± 88%	0.251	**0.08**^**b**^
7	2Nu + SDS	234 ± 49%	**0.025**^**a**^	412 ± 89%	**0.011**^**a**^	106 ± 15%	0.881	**0.016**^**a**^
8	5Nu	156 ± 30%	**0.053**^**b**^	490 ± 178%	**0.053**^**b**^	245 ± 66%	**0.053**^**b**^	**0.08**^**b**^
9	5Nu + SDS	165 ± 89%	0.456	384 ± 67%	**0.036**^**a**^	140 ± 76%	0.456	**0.014**^**a**^
10	10Nu	131 ± 57%	1	243 ± 153%	**0.045**^**a**^	171 ± 49%	0.699	0.56
11	10Nu + SDS	107 ± 6%	**0.014**^**a**^	8 ± 2%	**0.014**^**a**^	14 ± 11%	**0.014**^**a**^	**0.018**^**a**^

Results present as mean and SD. Statistical *P* values corresponding to the comparison of results obtained from each treatment with the untreated control.

*P values were calculated by ranking raw intensity data and Mann-Whitney U test.

**P values of multiple comparisons of global ECM components (collagens, glycoproteins and proteoglycans) between control and treatment group using Kruskal Wallis test with post-hoc Dunn-Bonferroni correction. *P* < 0.05 was considered statistically significance.

Significant *P* values are labeled with “a”, and marginally significant P values with “b”. Treatment abbreviations refer to Table 2.

**Table 2:** Decellularization conditions.

	**Treatment**	**Protocol with continuous agitation**
1	Control	In PBS
2	Hyperosmotic	(i) 1.5 M NaCl in PBS (24 h) * # § ς
3	Ionic detergent	(i) 0.1% SDS in PBS (24 h) * **# §**
4	Non-ionic detergent	(i) 0.1% Tx in PBS (24 h) **#**
5	Ionic & non-ionic detergents	(i) 0.1% SDS(ii) 0.1% Tx in PBS (24 h) Φ
6	Enzymatic (2Nu)	(i) 1.5 M NaCl in PBS (24 h),(ii) 2 U/ml nuclease in NaCl/PBS (24 h, twice)
7	Enzymatic & detergent (2Nu + SDS)	(i) 1.5 M NaCl in PBS (24 h),(ii) 2 U/ml nuclease in NaCl/PBS (24 h, twice),(iii) 0.1% SDS in PBS (24 h)
8	Enzymatic (5Nu)	(i) 1.5 M NaCl in PBS (24 h),(ii) 5 U/ml nuclease in NaCl/PBS (24 h, twice) **Φ ς**
9	Enzymatic & detergent (5Nu + SDS)	(i) 1.5 M NaCl in PBS (24 h),(ii) 5 U/ml nuclease in NaCl/PBS (24 h, twice),(iii) 0.1% SDS in PBS (24 h)
10	Enzymatic (10Nu)	(i) 1.5 M NaCl in PBS (24 h),(ii) 10 U/ml nuclease in NaCl/PBS (24 h, twice),
11	Enzymatic & detergent (10Nu + SDS)	(i) 1.5 M NaCl in PBS (24 h),(ii) 10 U/ml nuclease in NaCl/PBS (24 h, twice),(iii) 0.1% SDS in PBS (24 h)

*Reference 20; **#**Reference 31; **§**Reference 34; **Φ**Reference 21; **ς**Reference 22.

Note: NaCl, sodium chloride (Sigma-Aldrich); SDS, sodium dedecylsulfate (BioRad); Tx, Triton X-100 (Sigma); Nu, nuclease (DNase and RNase, Roche).
