# Supplementary material for: Decellularization of human stromal refractive lenticules for corneal tissue engineering
Source: Sci Rep. 2016 May 23;6:26339. doi: 10.1038/srep26339 (PMC4876320; doi:10.1038/srep26339)
Supplement: Supplementary Information [file srep26339-s1.pdf]

Decellularization of human stromal refractive lenticules for corneal tissue engineering

Gary Hin-Fai Yam, Nur Zahirah Binte M Yusoff, Tze-Wei Gwen, Melina Setiawan, Xiao-Wen Lee, Yu-Chi Liu & Jodhbir S Mehta

Supplementary Information

Supplementary Table 1. Donor cornea information

Donor cornea no.	Gender /Age	Time to Preservation	Cause of Death	Use in Figures
1	F/9	1 day	Head trauma	1
2	F/9	1 day	Head trauma	1
3	M/18	1 day	Motor vehicle accident	2, 3
4	M/18	1 day	Motor vehicle accident	2, 3
5	F/31	1 day	Motor vehicle accident	1, 2, 3
6	F/31	1 day	Motor vehicle accident	1, 2, 3
7	M/24	1 day	Motor vehicle accident	2, 3, 4
8	M/71	<1 day	Acute cardiac crisis	2, 3
9	M/71	<1 day	Acute cardiac crisis	2, 5, 6
10	M/24	1 day	Motor vehicle accident	2, 3, 4
11	F/22	<1 day	Overdose	2, 3
12	F/22	<1 day	Overdose	2, 6

Donor age ranged from 9 to 71 years old with a median age of 24 year old. The time taken from death of donor to preservation in Optisol GS was less than a day and the time to femtosecond laser cut was between 3 to 16 days.

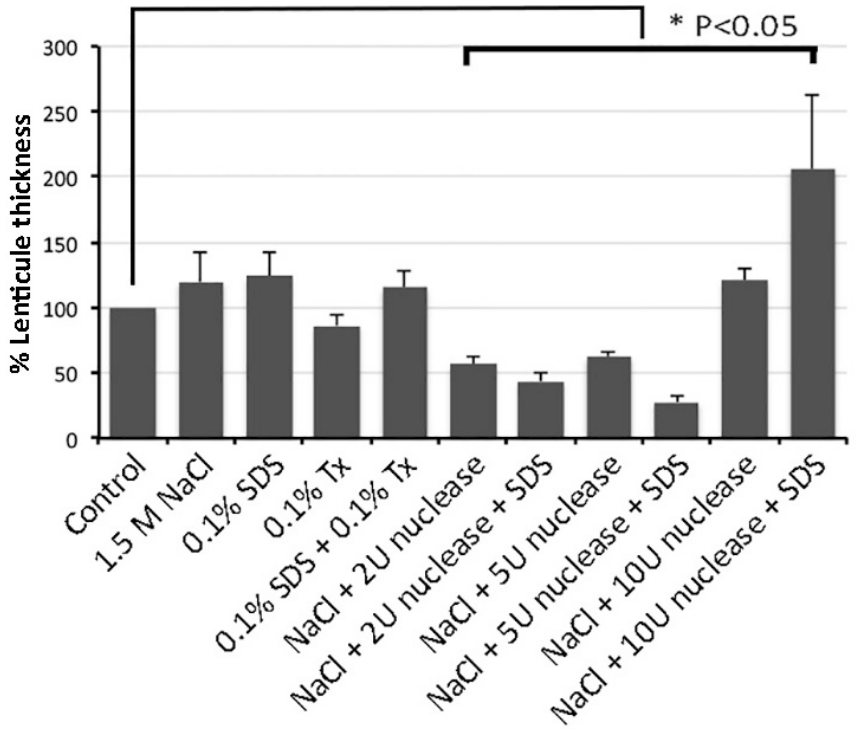

Supplementary Figure S1. Histogram showing lenticule thickness after decellularization. Data are presented as mean and SD and compared to control (100% thickness). Nuclease treatment resulted in significantly variable thickness (*P*<0.05, Mann-Whitney U test).

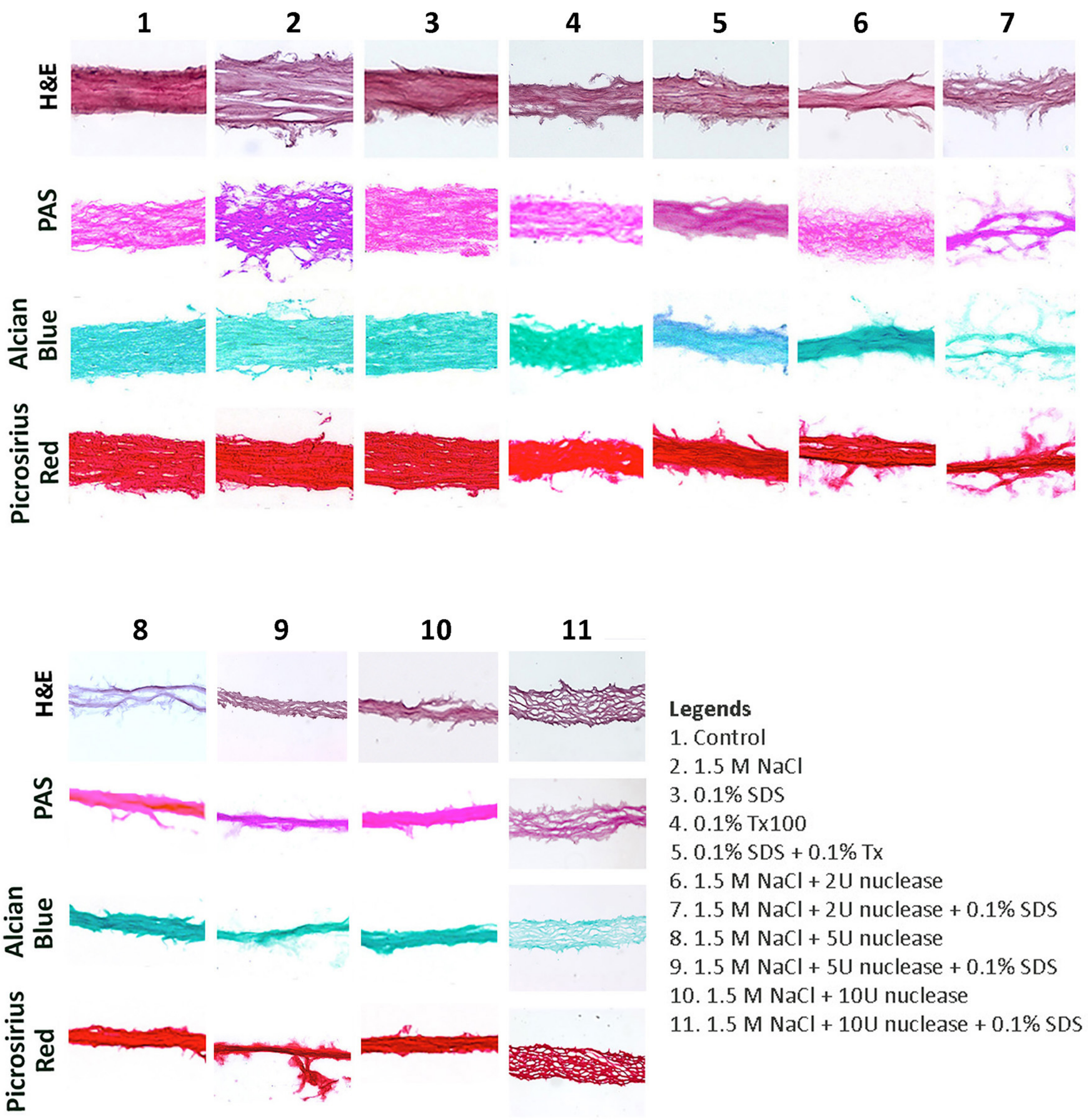

Supplementary Figure S2. Representative pictures of control and decellularized lenticules after histochemistry using hematoxylin-eosin (H&E), periodic acid-Schiff (PAS), alcian blue and picrosirius red staining.

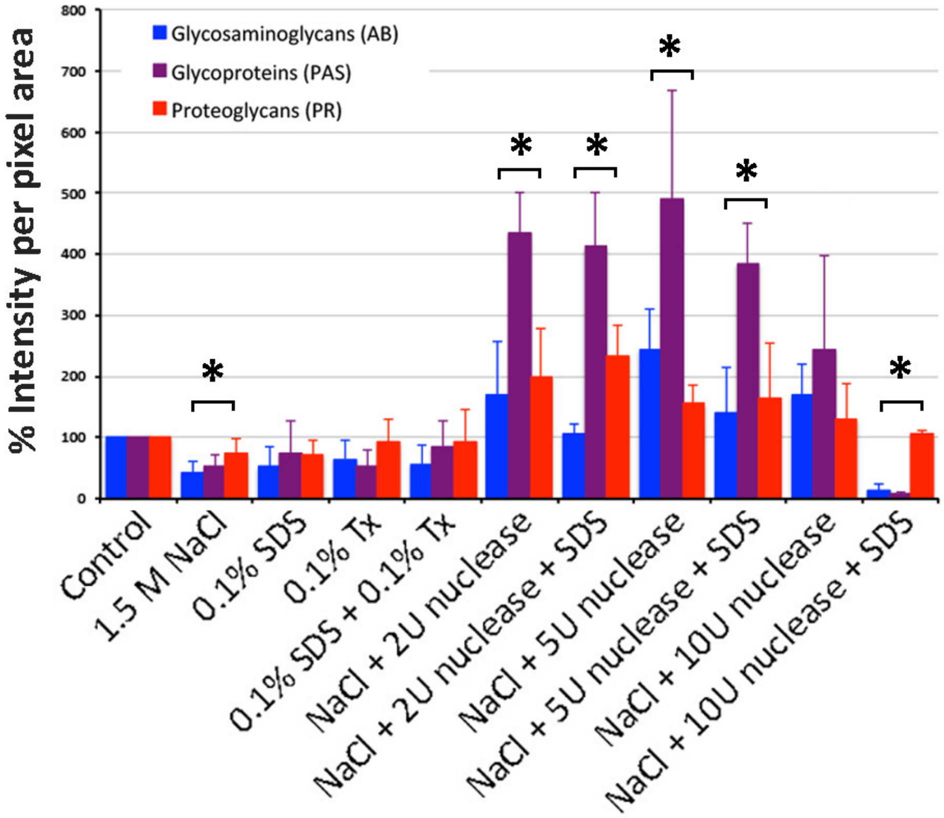

Supplementary Figure S3. Quantitative analysis of staining intensity of non-fibrillar ECM components (collagen, glycoprotein, GAGs) after histochemistry. **P*<0.05 for multiple comparisons of all 3 components between control and treatments.

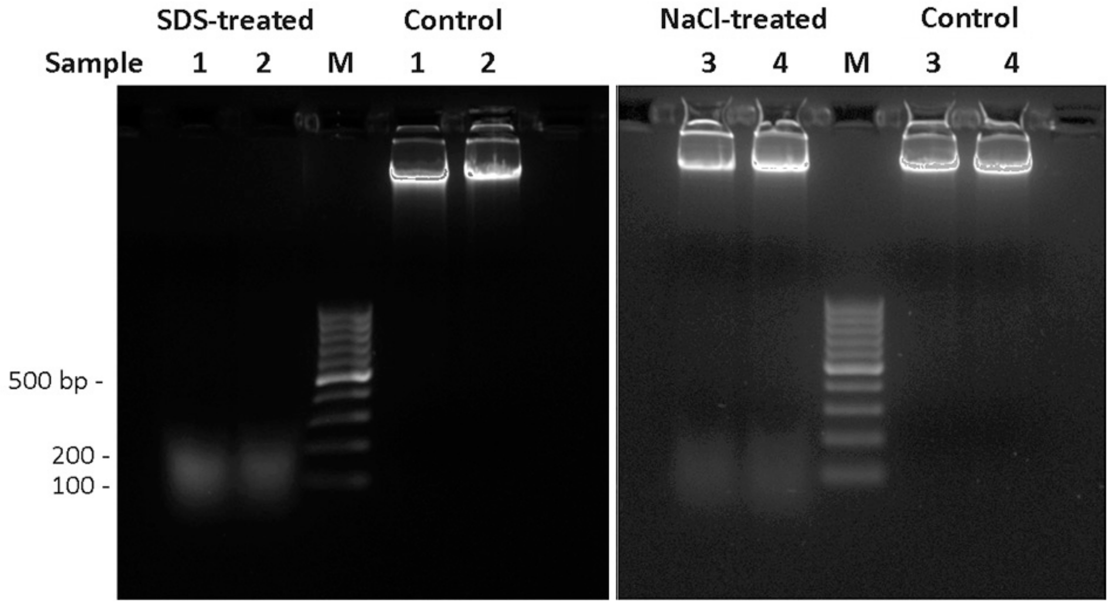

Supplementary Figure S4. Agarose gel electrophoresis of DNA extracted from decellularized lenticules. DNA fragments (predominantly <200 bp in size) were detected from SDS-treated lenticules whereas supercoiled DNA (>1 kb size) from control lenticules. NaCl-treated lenticules contained both <200 bp DNA fragments and supercoiled DNA.

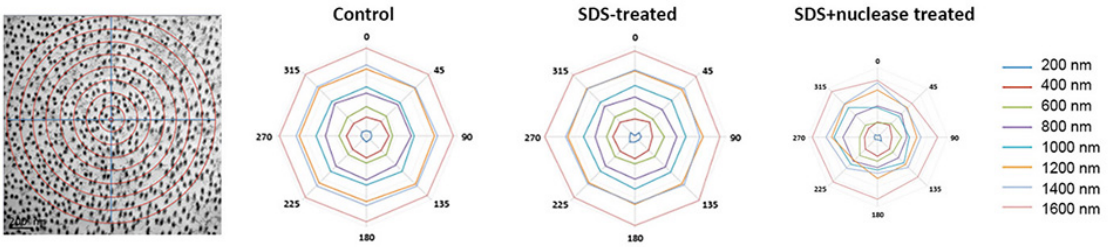

Supplementary Figure S5. Radial distribution function analysis of fibril organization at 360° pattern. On TEM images taken at 40,000x magnification, a reference fibril was randomly selected and overlaid with concentric circles with diameter increasing at 200 nm intervals and the number of fibrils was quantified in 8 quadrants (each spanning 45°) up to 1600 nm distance. Mean fibril numbers in different quadrants were represented with radar chart. Regular distribution pattern of collagen fibrils was observed in control and SDS-treated lenticules but not in 2N+SDS treated lenticles showing inconsistent fibril organization. Each group had n=6 sampling size. Scale bar: 200 nm.
